# Clinical and Microbiological Study on Local Application of an Ozonated Olive Oil Gel in the Periodontal Pockets: A Randomized Double-Blind Trial

**DOI:** 10.3390/jcm14155182

**Published:** 2025-07-22

**Authors:** Roberta Grassi, Fabio Ciccone, Domenico De Falco, Matteo Castaldi, Maria Teresa Agneta, Gianna Maria Nardi, Massimo Petruzzi

**Affiliations:** 1 Department of Oral Surgery, Tor Vergata University, 00100 Rome, Italy; grassi.roberta93@gmail.com; 2 School of Dentistry, Interdisciplinary Department of Medicine, University of Bari “Aldo Moro”, 70100 Bari, Italy; fabio.ciccone5@gmail.com (F.C.); agneta.mt.mta@gmail.com (M.T.A.); massimo.petruzzi@uniba.it (M.P.); 3 Academy of Advanced Technologies in Oral Hygiene Sciences (ATASIO), 70018 Rutigliano, Italy; castaldi.matteo@libero.it; 4 Department of Dental and Maxillofacial Sciences, “Sapienza” University of Rome, 00161 Rome, Italy

**Keywords:** oral health, periodontitis, olive oil, ozonated oil

## Abstract

**Objectives**: This study aims to evaluate the clinical and microbiological efficacy of a novel Activated Ozonated Extra-Virgin Olive Oil (AOEOO) gel as a topical adjunct in the treatment of periodontal pockets. **Methods**: In this double-blind, randomized clinical trial, patients diagnosed with stage II–IV periodontitis received either scaling and root planing (SRP) and placebo gel or SRP combined with subgingival AOEOO gel application (test group). Periodontal indices—probing pocket depth (PPD), clinical attachment level (CAL), plaque index (PI), and bleeding on probing (BOP)—were measured at baseline, 3, and 6 months. Microbiological analysis using real-time PCR quantified six key periodontal pathogens at baseline and after 6 months. **Results**: AOEOO-treated patients showed significantly greater improvements in PPD, CAL, PI, and BOP at both 3 and 6 months compared to the placebo group (*p* < 0.05). Also, microbiologically, the AOEOO group exhibited a significant reduction in total bacterial load and in all target pathogens, with reductions ranging from 63.8% to 98.7% (*p* < 0.05). No adverse effects were reported. **Conclusions**: The adjunctive use of AOEOO gel significantly improved periodontal outcomes and reduced pathogenic bacterial load, supporting its potential role as a safe and effective supportive treatment in periodontitis management.

## 1. Introduction

Periodontal disease remains one of the most prevalent chronic inflammatory conditions affecting adults worldwide, with significant implications for both oral and systemic health [1]. Periodontitis is characterized by the progressive destruction of tooth-supporting tissues, ultimately leading to tooth loss. A hallmark feature of advanced periodontitis is the formation of periodontal pockets, which serve as reservoirs for pathogenic bacteria and are challenging to manage effectively with conventional mechanical debridement alone [2].

Despite the widespread use of scaling and root planing (SRP) as the gold standard for nonsurgical periodontal therapy, its limitations in fully eradicating pathogenic biofilms—particularly in deep or inaccessible pockets—have prompted the exploration of adjunctive topical therapies. These therapies aim to enhance the decontamination of periodontal pockets, reduce inflammation, and promote tissue healing without the systemic side effects associated with antibiotics [3].

Currently available topical agents, such as chlorhexidine, doxycycline, and minocycline, have demonstrated clinical efficacy in reducing pocket depth and improving attachment levels. However, their use is not without drawbacks [4]. Most notably, chlorhexidine is associated with tooth staining, altered taste sensation, and mucosal irritation when used over extended periods. Tetracycline derivatives, while effective antimicrobials, carry the risk of bacterial resistance and may disrupt the local microbial ecology, potentially impairing long-term periodontal health [5].

Furthermore, the physicochemical properties of many conventional topical formulations often limit their retention within the periodontal pocket. Rapid clearance by gingival crevicular fluid or insufficient penetration into biofilms can significantly reduce therapeutic effectiveness [6]. These limitations highlight the need for alternative topical agents that not only possess strong antimicrobial and anti-inflammatory properties but also offer improved bioadhesion, biocompatibility, and sustained release characteristics [7].

In this context, ozonized olive oil-based gels are emerging as a promising new class of topical therapeutics for periodontal applications. Ozone, a triatomic form of oxygen (O_3_), is recognized for its potent antimicrobial, anti-inflammatory, and immunostimulatory effects. The use of ozone in inflammation management has gained increasing attention in recent years due to its anti-inflammatory and immunomodulatory potential [8]. When administered at controlled concentrations, ozone exerts its therapeutic effects primarily through the modulation of oxidative stress and the activation of antioxidant defense mechanisms [9]. Ozone interacts with biological membranes, leading to the production of reactive oxygen species (ROS) and lipid oxidation products (LOPs), which act as signaling molecules. These molecules can modulate the immune response by reducing the release of pro-inflammatory cytokines such as TNF-α, IL-1β, and IL-6, while promoting the production of anti-inflammatory mediators like IL-10 [10].

Several studies have demonstrated that ozone therapy can improve local microcirculation, reduce tissue hypoxia, and decrease inflammatory cell infiltration [11]. The anti-inflammatory effects of ozone are also associated with modulation of the NF-κB pathway, which plays a crucial role in regulating inflammation [12]. Despite its promising potential, ozone therapy must be administered within precise therapeutic windows to avoid cytotoxic effects associated with excessive oxidative stress [13].

Ozone is also a powerful antimicrobial agent with well-documented antibacterial, antiviral, and antifungal properties. Its antibacterial effect is primarily mediated through its strong oxidative potential, which leads to the disruption of bacterial cell walls and membranes [14]. This oxidative stress increases membrane permeability, causes leakage of cellular contents, and ultimately results in bacterial cell death [15]. Unlike traditional antibiotics, ozone acts non-selectively on microbial structures, reducing the risk of developing antimicrobial resistance [16].

Studies have shown that ozone is effective against a broad spectrum of bacteria, including both Gram-positive and Gram-negative strains, as well as antibiotic-resistant pathogens. Its mechanism involves the oxidation of phospholipids and lipoproteins in the cell envelope, along with damage to intracellular components such as enzymes and nucleic acids [17,18].

Ozone’s bactericidal effect is achieved without harming surrounding healthy tissues when applied at controlled therapeutic concentrations, representing a promising complementary tool in infection control—particularly in the era of rising antibiotic resistance [19].

When incorporated into a lipid carrier such as olive oil, ozone’s stability and ease of application are markedly enhanced. Ozonized olive oil can be formulated into a gel with ideal rheological properties for insertion into periodontal pockets, allowing for prolonged contact with the target site [20].

Olive oil itself is rich in monounsaturated fatty acids and phenolic compounds, which confer antioxidant and anti-inflammatory benefits. These properties may further support periodontal tissue healing and help reduce oxidative stress within the periodontal environment. Additionally, the natural origin and biocompatibility of olive oil minimize the risk of adverse tissue reactions, making it suitable for repeated applications [21].

Several in vitro and in vivo studies have demonstrated the antimicrobial efficacy of ozonized oils against periodontopathogenic microorganisms, including *Porphyromonas gingivalis*, *Aggregatibacter actinomycetemcomitans*, and *Fusobacterium nucleatum*. Preliminary clinical trials suggest improvements in probing depth, bleeding on probing, and clinical attachment levels when ozonized oils are used adjunctively with mechanical debridement [20]. However, comprehensive clinical data remain limited, and standardized protocols regarding dosage, frequency, and duration of application have yet to be established.

The purpose of this trial is to evaluate the clinical efficacy and safety of a novel ozonized olive oil-based gel as a topical adjunct in the treatment of periodontal pockets. Specifically, this research aims to assess its impact on clinical periodontal indices and microbial load.

## 2. Materials and Methods

### 2.1. Study Design, Ethics, and Participants

This single-center, double-blind, randomized clinical and microbiological trial was conducted in accordance with the CONSORT-Outcomes 2022 extension statement. The study aimed to investigate the clinical and microbiological effects of locally applied Activated Ozonated Extra-Virgin Olive Oil (AOEOO) in the management of periodontal pockets over a 6-month period. The study was registered at ClinicalTrials.gov (ID: NCT06980675, 11 January 2024). Ethical approval was obtained from the Local Ethical Review Board in Bari (Study No. 7493, Prot. No. 0015250, 20 January 2021), and the study adhered to both institutional and international ethical standards, including the Declaration of Helsinki. All participants provided written informed consent.

Consecutive patients with a preliminary diagnosis of periodontitis, confirmed by a specialist periodontist, were recruited from the Dental Clinic at the University of Bari “Aldo Moro” (Southern Italy) between 2023 and 2024.

### 2.2. Inclusion and Exclusion Criteria

Inclusion criteria were as follows:

(i) Diagnosis of stage II, III, or IV periodontitis, grade B or C (according to the 2017 World Workshop on the Classification of Periodontal and Peri-Implant Diseases and Conditions) [22];
(ii) Presence of at least one quadrant with a minimum of five teeth that had not undergone periodontal debridement in the previous 12 months;
(iii) Probing pocket depth (PPD) ≥ 4 mm at two or more sites in different quadrants;
(iv) Age ≥ 18 years;
(v) No use of systemic medications;
(vi) Signed informed consent;
(vii) Demonstrated compliance with the study protocol.

Exclusion criteria were as follows:

(i) Pregnant or lactating women, or women of childbearing potential not using reliable contraception;
(ii) Use of systemic antibiotics or corticosteroids within 3 months prior to the study;
(iii) Known allergy to any component of ozonated olive oil;
(iv) Current smokers.

### 2.3. Randomization and Patient Allocation

Randomization was performed using an online random sequence generator (https://www.randomlists.com/random-letters, accessed on 11 November 2020), assigning each patient a unique identifier linked to either Group A (Placebo) or Group B (AOEOO gel).

The sample size calculation was based on the study by Patel et al. [23], using the mean and standard deviation (SD) of the clinical attachment level (CAL) as the primary outcome. Assuming a significance level of 5% and a power of 80% (calculated using OpenEpi, http://www.openepi.com), the required sample size was 32 sites.

A single operator performed all treatments, including gel application and clinical measurements. Both the operator and the outcome assessor were blinded to the group assignments to minimize bias.

### 2.4. Clinical Outcomes

The primary outcome was the Clinical Attachment Level (CAL), defined as the distance from the cementoenamel junction (CEJ) to the bottom of the gingival sulcus. Probing Pocket Depth (PPD) was measured as the distance from the gingival margin to the base of the pocket.

Measurements were recorded at six sites per tooth, namely, mesiobuccal, buccal, distobuccal, mesiolingual, lingual, and distolingual, using a UNC-15 periodontal probe (Nordent Manufacturing Inc., Elk Grove Village, IL, USA).

Bleeding on Probing (BOP) was assessed for the entire mouth using a dichotomous score (yes/no) according to O’Leary et al. [24]. The Plaque Index (PI) was recorded according to Löe and Silness [25].

### 2.5. Operative Phase

All patients received comprehensive nonsurgical periodontal therapy in a single session, including SRP (Scaling and Root Planing) to remove both hard and soft deposits from all teeth.

Subgingival debridement was performed under local anesthesia using ultrasonic scalers (Woodpecker UDS-P with LED, China) and hand instruments, including Gracey curettes, After-Five Gracey curettes, and universal curettes (Hu-Friedy, Chicago, IL, USA).

Oral hygiene instructions and biofilm control were reinforced during follow-up, including supragingival plaque removal and re-instruction on oral hygiene techniques. Patients were instructed to report any discomfort or side effects after treatment.

Patients in the test group (Group B) received SRP plus subgingival application of AOEOO gel (Ialozon Gel^®^, Gemavit SRL, Elmas (CA), Italy), delivered using a 3 mL disposable plastic syringe with a blunt 23-gauge needle. Following SRP, treated teeth were isolated with cotton rolls and gently dried. Approximately 1–1.5 mL of AOEOO gel was applied subgingivally and interproximally until slight excess was visible at the gingival margin, which was then removed with a cotton roll.

No antiplaque agents, systemic antibiotics, or anti-inflammatory drugs were prescribed. Additionally, patients were advised to avoid using any dental cleaning aids that might interfere with the gel application site for at least 2 days after treatment.

### 2.6. Gel and Placebo Composition

The placebo gel used for Group A consisted of water, glycerin, VP/VA copolymer, polysorbate 80, potassium sorbate, sodium benzoate, lactic acid, and disodium EDTA.

The active gel used for Group B contained AOEOO, cetylpyridinium chloride, chlorphenesin, low-molecular-weight collagen, and both low- and high-molecular-weight hyaluronic acid.

The two gels were identical in appearance, consistency, and taste, making them indistinguishable to both patients and the operator. Patients were instructed not to rinse their mouths or eat for at least 30 min after gel application.

### 2.7. Microbiological Assessment

Microbiological samples were collected by inserting three sterile paper points subgingivally into the three deepest periodontal pockets until resistance was met and left in place for 20 s.

The tips of two paper points were then cut with sterile scissors and placed into sterile vials. This procedure was performed at baseline and again at the 6-month follow-up.

Samples were transported to the laboratory and processed using the Applied Biosystems 7500 Real-Time PCR System (Thermo Fisher Scientific, Waltham, MA, USA), following the manufacturer’s instructions.

Six bacterial taxa were identified according to Papapanou et al. [26]: *Aggregatibacter actinomycetemcomitans*, *Porphyromonas gingivalis*, *Tannerella forsythia*, *Treponema denticola*, *Fusobacterium nucleatum*, and *Campylobacter rectus*.

Results were expressed as bacterial copy counts for each microorganism.

### 2.8. Study Timeline

Follow-up visits were scheduled at 3 and 6 months postoperatively for clinical assessments. Microbiological analysis was performed at baseline and at 6 months (study completion).

### 2.9. Statistical Analysis

Descriptive statistics were reported for patient demographics (age, sex) and baseline periodontal parameters. Clinical outcomes were expressed as means and standard deviations (SD) for quantitative variables.

Comparisons between groups were performed using either the Student’s *t*-test (for normally distributed data) or the Wilcoxon–Mann–Whitney test (for non-normally distributed data). The effects of potential confounding factors such as age, sex, and baseline periodontal status were assessed using multiple linear regression models.

Significance was set at *p* < 0.05. Data analysis was conducted using Microsoft Excel.

**Figure 1:**
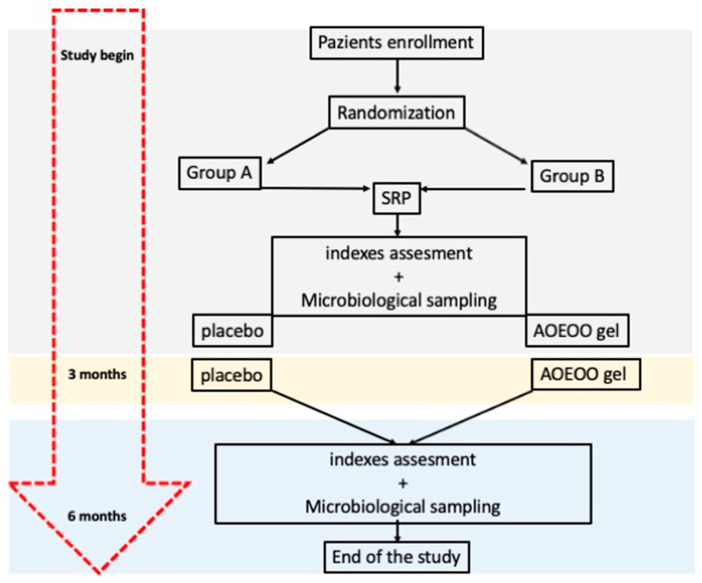
Study flow chart.

A summary of the study flow is presented in Figure 1.

## 3. Results

Twenty-seven patients with a mean age of 48 ± 7 years were enrolled, and forty periodontal pockets were analyzed. The male–female ratio was 5:7, with no differences in the two groups with respect to sex and age.

Table 1 reports the data relative to the clinical indices recorded in both groups before and after the gel treatment (placebo and AOEOO) and their comparison after 3 and 6 months.

In the AOEOO group, PPD showed a statistically significant reduction as early as 3 months, whereas in the placebo group, a significant decrease in PPD was observed only at 6 months. The improvement in PPD was significantly greater and more rapid (*p* < 0.05) in patients treated with AOEOO.

Similar results were observed for CAL, which improved both more significantly and more rapidly in the AOEOO-treated group. PI scores were also significantly better (*p* < 0.05) when AOEOO gel was applied to the periodontal pockets. Furthermore, a marked reduction in gingival bleeding was observed in the periodontal sites of patients in Group B.

Microbiological data also demonstrated a significant reduction in bacterial load in patients treated with AOEOO compared to the placebo group.

At baseline, there were no statistically significant differences between the groups for any of the bacterial parameters, including total bacterial load and specific pathogens such as *Aggregatibacter actinomycetemcomitans*, *Porphyromonas gingivalis*, *Treponema denticola*, *Tannerella forsythia*, *Fusobacterium nucleatum*, and *Campylobacter rectus* (*p* > 0.05 for all comparisons).

After six months, Group B exhibited a significant reduction in total bacterial load, decreasing from 16,410,000 ± 20,500,000 to 9,750,000 ± 12,800,000 bacterial copy counts, corresponding to a 40.6% reduction (*p* = 0.03).

In contrast, Group A showed a non-significant increase from 16,082,024 ± 21,044,706 to 17,638,983 ± 28,769,619 bacterial copy counts (*p* = 0.61).

The between-group comparison at six months revealed a statistically significant difference in favor for the treatment group (*p* = 0.04).

Regarding specific periodontal pathogens, Group B demonstrated significant reductions in *Aggregatibacter actinomycetemcomitans* (63.8% reduction, *p* = 0.02), *Porphyromonas gingivalis* (76.2% reduction, *p* = 0.01), *Treponema denticola* (98.7% reduction, *p* = 0.002), *Tannerella forsythia* (86.5% reduction, *p* = 0.01), *Fusobacterium nucleatum* (64.7% reduction, *p* = 0.03), and *Campylobacter rectus* (97.7% reduction, *p* = 0.001).

In contrast, Group A did not exhibit significant changes in the levels of these pathogens over the same period (*p* > 0.05 for all comparisons).

The microbiological findings are summarized in Table 2.

Multiple regression analysis showed that age, sex, and baseline periodontal status did not significantly affect the study outcomes.

No side effects or adverse reactions were recorded during the study.

## 4. Discussion

The application of AOEOO gel as an adjunctive treatment to SRP significantly reduced both total bacterial load and specific periodontal pathogens compared to the placebo, demonstrating promising efficacy in periodontitis management.

Ozone therapy is an effective tool in the treatment of periodontal diseases. The combination of ozone and olive oil generates molecules such as ozonides, aldehydes, peroxides, and hydroxyperoxides, which enhance its antimicrobial activity. Therefore, the association of these two substances represents a valuable and cost-effective resource in the management of periodontal disease [27].

The findings of our study demonstrated a statistically significant reduction in PPD following the application of a gel formulated with Activated Ozonated Extra-Virgin Olive oil. Additionally, a substantial reduction in bacterial load was observed post-treatment, specifically in key periodontopathogenic species, including *Aggregatibacter actinomycetemcomitans*, *Porphyromonas gingivalis*, *Tannerella forsythia*, *Treponema denticola*, *Fusobacterium nucleatum*, and *Campylobacter rectus*.

The reduction in the periodontal pathogenic bacterial load may induce a microbiological shift towards non-periodontopathogenic species, which could occupy the ecological niche left vacant. These new colonizers do not release cytotoxic metabolites and do not trigger an immune-inflammatory response—both key factors in the pathogenesis of periodontal disease. This hypothesis, supported by the studies of Berezov et al. [28], could also explain the clinical improvements observed, likely associated with a reduced reactive-inflammatory state of the periodontium. A similar concept was previously explored by Baysan et al., who demonstrated that ozone therapy significantly reduced the number of Gram-positive bacteria in carious root lesions, leading to clinical improvements and arrest of lesion progression [29].

The efficacy of ozone against Gram-positive and Gram-negative bacteria, viruses, and fungi was also investigated by Nagayoshi et al., who treated periodontopathogenic bacteria in vitro with ozonated water. Their study demonstrated antimicrobial activity against *Streptococcus*, *Porphyromonas gingivalis*, *Porphyromonas endodontalis*, *Aggregatibacter actinomycetemcomitans*, and *Candida albicans*, both in planktonic cultures and biofilms. The authors concluded that Gram-negative anaerobes, such as *P. endodontalis* and *P. gingivalis*, were substantially more susceptible to ozonated water compared to Gram-positive *oral streptococci* and *C. albicans* in pure culture [30].

Moreover, some authors have observed greater reductions in PI and gingival index when comparing ozone irrigation to chlorhexidine. These findings suggest a potentially more significant role of ozone in microbial control compared to chlorhexidine [31].

Katti et al. demonstrated that ozonated water was effective in reducing the microbial load of Gram-negative bacteria such as *Porphyromonas gingivalis*, *Prevotella intermedia*, and *Fusobacterium nucleatum*, as well as in decreasing the PI [32].

The study by Hayakumo et al. also reported reductions in PPD, increases in CAL, and significant decreases in bacteria within subgingival plaque. This further highlights the potential of ozone as an effective antiseptic agent as its transformation into oxygen leads to the generation of hydroxyl radicals, which are among the most reactive oxidative species. These free radicals may therefore play a key role in bacterial destruction [33].

However, clinical studies, such as those by Müller et al. and Seydanur Dengizek et al., have reported conflicting results regarding the use of ozonated substances applied within periodontal pockets. These studies did not demonstrate any significant improvements in clinical parameters when ozone was used in combination with scaling and root planing [34,35].

Natural oils are known to possess various beneficial properties, including neuroprotective, anti-inflammatory, and antimicrobial effects [36]. Specifically, the combination of ozone with olive oil in the treatment of periodontitis has been evaluated in several studies. Puxeddu et al. [37] investigated the antimicrobial properties of natural oils, particularly ozonated olive oil and ozonated sunflower seed oil, focusing on the biological effects of ozone dissolved in natural oils. Their results showed very high antimicrobial activity, especially against *Candida albicans* and *Enterococcus faecalis*, moderate activity against *Staphylococcus aureus* and *Escherichia coli*, and low or nearly absent activity against *Pseudomonas aeruginosa* and *Klebsiella pneumoniae* [37].

In the study conducted by Mukherjee et al., the clinical efficacy of ozonated olive oil, plain olive oil, and chlorhexidine gel was evaluated as adjuncts to SRP in patients with chronic periodontitis. Both ozonated olive oil and chlorhexidine gel demonstrated significantly greater improvements in periodontal health compared to plain olive oil. Ozonated olive oil was applied locally as a drug delivery system in conjunction with SRP. The authors reported a statistically significant reduction in gingival index, PPD, and CAL, with no observed side effects and good patient tolerance [20].

A recent meta-analysis by D’Ambrosio et al. [38] extensively discussed the adjunctive effect of ozone therapy in the management of periodontitis and peri-implantitis. This study analyzed various approaches, including the use of ozonated olive oil combined with SRP, and demonstrated improvements in clinical parameters such as BOP and PPD, as also reported by Patel and Nardi [23,39]. Patel et al. highlighted that the subgingival application of topical ozonated olive oil as an adjunct to SRP significantly improved both BOP and PPD in patients with chronic periodontitis [23]. Similarly, Nardi et al. demonstrated that adding a mouthwash based on ozonated olive oil to nonsurgical periodontal therapy resulted in a more effective reduction in salivary MMP-8 levels compared to nonsurgical therapy alone, suggesting a benefit in modulating the inflammatory response within the periodontium [39].

However, not all studies have reported consistent findings. Notably, Nambiar et al. observed no statistically significant differences in BOP and PPD outcomes between SRP treatments supplemented with ozonated olive oil and those using chlorhexidine. This study provided no clinical evidence to suggest a superior benefit of ozonated olive oil over chlorhexidine as an adjunct to nonsurgical periodontal therapy [40]. Similarly, Gandhi et al. reported no significant differences between ozonated olive oil and chlorhexidine in terms of improvements in clinical parameters, including plaque index, gingival index, PPD, CAL, or reductions in periodontal pathogenic bacterial counts [41].

These contrasting results suggest that while ozonated olive oil may offer benefits in reducing inflammation and promoting periodontal healing, it does not consistently lead to substantially greater clinical improvements compared to conventional treatments such as chlorhexidine.

This study did not include microbiological analysis to assess changes in pathogenic bacteria, and the evaluation of inflammatory markers before and after AOEOO administration could be considered in future research. Additional limitations are the lack of assessment regarding patient satisfaction with the gel and the monocentric nature of the trial, as it was conducted in a single center. Nevertheless, it is important to highlight that no side effects related to the administration of either the gel or the placebo were reported by any patient enrolled in the study.

The present study is intended as a pilot, and based on the results obtained, it may serve as a starting point for further investigations. Ozone therapy—particularly in the form of ozonated olive oil—may represent a valuable adjunct in periodontal therapy; however, further studies are required to confirm its long-term efficacy and potential superiority over conventional treatments. AOEOO gel demonstrated effectiveness as an adjunctive treatment for periodontal pockets, leading to improvements in periodontal indices and reductions in bacterial load.

## Figures and Tables

**Table 1:** Comparison between clinical periodontal indices in groups A and B.

Indices	Group	Baseline Value (Mean)	After 3 Months (Mean)	After 6 Months (Mean)	*p*-Value Intra-Group (3 Months)	*p*-Value Intra-Group (6 Months)	*p*-Value Inter-Group (3 Months)	*p*-Value Intra-Group (6 Months)
**PPD (mm)**	A	4.2	4.0	3.9	0.08	0.045	0.04	0.001
	B	4.3	3.5	3.2	0.02	0.001		
**CAL (mm)**	A	4.9	4.7	4.6	0.07	0.043	0.03	0.001
	B	5.1	4.0	3.6	0.01	0.001		
**PI (0–3)**	A	1.6	1.5	1.4	0.09	0.045	0.03	0.001
	B	1.6	1.1	0.9	0.02	0.001		
**BOP (0–1)**	A	0.6	0.5	0.5	0.10	0.046	0.03	0.001
	B	0.7	0.4	0.3	0.01	0.001		

PPD: periodontal probing depth; CAL: clinical attachment level; PI: plaque index, BOP: beading on probing. Group A: placebo gel; Group B: activated ozonated EVO olive oil gel.

**Table 2:** Microbiological data.

	Group	Initial (Mean ± SD)	After 6 Months (Mean ± SD)	*p* Intra-Group	*p* Inter-Group (Final)
**Total bacterial load** (bacterial copy count number)	A	16,082,024 ± 21,044,706	17,638,983 ± 28,769,618	0.61	**0.04**
	B	16,410,000 ± 20,500,000	9,750,000 ± 12,800,000	**0.03**	
***Aggregatibacter actinomycetemcomitans***	A	510,000 ± 910,000	570,000 ± 850,000	0.48	**0.03**
	B	525,000 ± 905,000	190,000 ± 300,000	**0.02**	
***Porphyromonas gingivalis***	A	632,645 ± 988,973	985,515 ± 1,200,000	0.18	**0.03**
	B	610,000 ± 950,000	145,000 ± 230,000	**0.01**	
***Treponema denticola***	A	1,200,000 ± 1,500,000	1,310,000 ± 1,550,000	0.56	**0.001**
	B	1,230,000 ± 1,600,000	15,500 ± 20,000	**0.002**	
***Tannerella forsythia***	A	224,236 ± 500,000	240,000 ± 510,000	0.67	**0.02**
	B	230,000 ± 505,000	31,000 ± 50,000	**0.01**	
***Fusobacterium nucleatum***	A	1,800,000 ± 2,300,000	2,000,000 ± 2,500,000	0.40	**0.04**
	B	1,900,000 ± 2,400,000	670,000 ± 900,000	**0.03**	
***Campylobacter rectus***	A	320,000 ± 510,000	310,000 ± 520,000	0.88	**0.001**
	B	330,000 ± 500,000	7500 ± 12,000	**0.001**	

Bold: significant values. Group A: placebo gel; Group B: activated ozonated EVO olive oil gel.

## Data Availability

The data supporting the findings of this study are available upon reasonable request by contacting the corresponding author at defalcodomenico@ymail.com.
